# Angiotensin-Converting Enzyme ID Polymorphism in Patients with Heart Failure Secondary to Chagas Disease

**DOI:** 10.5935/abc.20170137

**Published:** 2017-10

**Authors:** Silene Jacinto da Silva, Salvador Rassi, Alexandre da Costa Pereira

**Affiliations:** 1 Ciências da Saúde - Faculdade de Medicina - Universidade Federal de Goiás, Goiânia, GO – Brazil; 2 Instituto do Coração (Incor) do Hospital das Clínicas da Faculdade de Medicina da Universidade de São Paulo (HCFMUSP), São Paulo, SP - Brazil

**Keywords:** Chagas Disease, Polymorphism, Genetic, Heart Failure, Chagas Cardiomyopathy

## Abstract

**Background:**

Changes in the angiotensin-converting enzyme (ACE) gene may contribute to the increase in blood pressure and consequently to the onset of heart failure (HF). The role of polymorphism is very controversial, and its identification in patients with HF secondary to Chagas disease in the Brazilian population is required.

**Objective:**

To determine ACE polymorphism in patients with HF secondary to Chagas disease and patients with Chagas disease without systolic dysfunction, and to evaluate the relationship of the ACE polymorphism with different clinical variables.

**Methods:**

This was a comparative clinical study with 193 participants, 103 of them with HF secondary to Chagas disease and 90 with Chagas disease without systolic dysfunction. All patients attended the outpatient department of the General Hospital of the Federal University of Goias general hospital. Alleles I and D of ACE polymorphism were identified by polymerase chain reaction of the respective intron 16 fragments in the ACE gene and visualized by electrophoresis.

**Results:**

In the group of HF patients, 63% were male, whereas 53.6% of patients with Chagas disease without systolic dysfunction were female (p = 0,001). The time from diagnosis varied from 1 to 50 years. Distribution of DD, ID and II genotypes was similar between the two groups, without statistical significance (p = 0,692). There was no difference in clinical characteristics or I/D genotypes between the groups. Age was significantly different between the groups (p = 0,001), and mean age of patients with HF was 62.5 years.

**Conclusion:**

No differences were observed in the distribution of (Insertion/Deletion) genotype frequencies of ACE polymorphism between the studied groups. The use of this genetic biomarker was not useful in detecting a possible relationship between ACE polymorphism and clinical manifestations in HF secondary to Chagas disease.

## Introduction

Chagas disease has characteristics of an endemic disease and is an important cause of dilated heart disease and heart failure (HF) in regions of low socioeconomic level, leading to high mortality and morbidity rates. Early diagnosis and treatment are important to improve survival rates and quality of life.^1^

Chagas disease was considered the main cause of HF in central-western region of Brazil.^2,4^ Sudden cardiac death affects approximately 50% of patients with HF secondary to Chagas disease.^5^

Most human health problems, including HF, have a multifactorial etiology, influenced by environmental and genetic factors, and life style. Multifactorial disorders are characterized by phenotypic contributions of several genes that interact to each other and to environmental factors. Many disorders manifested in adults are inherited in an autosomal dominant fashion, including familial cardiomyopathy.^6^

Angiotensin-converting enzyme (ACE) gene (21 kb) is located in the chromosome 17, long arm, region 23, and contains 24 introns.^7^ It would be ideal to predict the individual response to therapy as well as the potential adverse effects of drugs in the treatment of HF. With advances in molecular biology and genetics, there has been an increasing need for a redefinition of diseases based on their biochemical processes rather than their phenotypic features. Hence, knowledge and treatment of heart diseases, and isolation and characterization of the genes involved may not be a panacea anymore, but rather, a starting point for an individualized treatment.

In this context, the present study aimed to determine the distribution of the ACE gene polymorphism (I/D) in HF secondary to Chagas Disease, compare it with that in Chagas Disease patients free of systolic dysfunction, and evaluate its relationship with clinical variables.

### Ethical aspects

The study was analyzed and approved by the Research Ethics Committee of the General Hospital of the Federal University of Goias on December 16^th^, 2014 (approval number 908870).

### Study design

This was a comparative, clinical study conducted with two groups of patients (group A and group B) attending the cardiology and the Chagas disease outpatient clinics of the General Hospital of the Federal University of Goias. Patients were recruited to the study from February 2014 to October 2015.

### Patients

A total of 193 outpatients were consecutively recruited, 103 with chagasic heart disease (group A) and 90 Chagas disease patients without systolic dysfunction (group B).

### Inclusion criteria

Group A: patients with symptomatic HF (according to Framingham criteria) secondary to Chagas disease; group B: patients with diagnosis of Chagas disease, free of systolic dysfunction.

### Exclusion criteria

Cardiac dysfunction in group B.

### Clinical and laboratory parameters

All clinical data were collected from patients' medical records. Recent laboratory, echocardiography and Doppler echocardiography results were used to determine patients' current health status.

In HF patients, functional class was determined using the New York Heart Association criteria, by the outpatient medical staff. With respect to Doppler echocardiography, the following parameters were analyzed: left atrium (LA), left ventricular systolic diameter (LVSD), left ventricular diastolic diameter (LVDD), and left ventricular ejection fraction (LVEF).

### Genotyping

Eight-mL blood samples were collected and stored in two tubes containing EDTA anticoagulant. Then, DNA extraction was performed, followed by ACE polymorphism genotyping by polymerase chain reaction (PCR), which was classified as D/D (deletion/deletion), I/D (insertion/deletion) or I/I (insertion/insertion).

Genotyping method was adapted from Lindpaintner et al.^8^ For a final volume of 25 µL, 1 mM of primers, 200 mM of nucleoside triphosphates (dATP, dCTP, dGTP, dTTP), 1.3 mM of magnesium chloride, 50 mM of potassium chloride, 0.5 unit of Taq DNA polymerase and 20 ng of DNA were added. The sense primer GCCCTGCAGGTGTCTGCAGCATGT and the antisense primer GGATGGCTCTCCCCGCCTTGTCTC were used to amplify the alleles D and I, resulting in amplicons of 319 pb and 597 pb, respectively. The protocol of DNA amplification was composed of an initial denaturation at 94ºC for 5 minutes, followed by 35 cycles - 30 seconds at 94ºC, 45 seconds at 56ºC, 2 minutes at 72ºC. Then, the amplification products of D and I alleles were subjected to 1.5% agarose gel electrophoresis stained with 0.5 mg/mL ethidium bromide for 10 minutes. Due to the preferential amplification of D allele in heterozygous samples, all samples with a DD genotype were reanalyzed using the primers TGGGACCACAGCCGCGCCTACCAC and TCGGCCCTCCCACCACCATGCTAA (sense and antisense, respectively), at the same conditions of PCR, except for the annealing temperature of 67ºC. Analysis of the PCR products by 1.5% agarose gel electrophoresis revealed an amplicon of 335pb with the allele I. The results were captured using the Image Master VDS® video documentation system (Pharmacia Biotech, EUA).

### Statistical analysis

Descriptive analysis was used for characterization of the variables - categorical variables were described in percentages; continuous variables with normal distribution were described in mean ± standard deviation, and continuous variables without normal distribution were described in median and interquartile ranges. The Kolmogorov-Smirnov Z test was used to identify those variables with a normal distribution. Differences between groups A and B were calculated using the chi-square test or the unpaired Student's t-test, and the Mann-Whitney test as appropriate. The association between the variables of exposure to HF was measured by Odds Ratio (OR) and respective 95% confidence intervals. Differences between the groups were considered statistically significant when p < 0.05. Analyses were performed using the SPSS program, version 18.0.

## Results

There was a significant difference in sex distribution between the groups (p = 0.023), and 63% of HF patients were men. Mean age of HF patients was 62.5 years ± 11.1 years, with significant difference between the groups (p = 0.00). Sociodemographic and clinical characteristics of patients are described in Table 1.

**Table 1:** Sociodemographic and clinical characteristics of the sample

Variables	Group A		Group B		OR	95CI%	p-value
	n	%	n	%			
**Sex**							
Male	51	63.0	30	37.0	1.96	1.09-3.52	0.023^a^
Female	52	46.4	60	53.6			
Mean age (SD)	62.5	(11.1)	51.3	(11.9)			0.000^b^
**Origin**							
Goiania	59	54.6	49	45.4	1.12	0.64-1.98	0.692^a^
Others	44	51.8	41	48.2			
Median time elapsed from diagnosis of Chagas disease****(interquartile range)	15	(8-25)	9.5	(5-17)			0.002^c^
**Smoking**							
Yes	30	73.2	11	26.8	2.95	1.38-6.32	0.004^a^
No	73	48.0	79	52.0			
**Alcohol consumption**							
Yes	21	42.0	29	58.0	0.34	0.28-1.03	0.061^a^
No	82	57.3	61	42.7			
Median heart rate (interquartile range) (bpm)	65	(60-80)	65	(60-80)			0.290^c^
**Megaesophagus**							
Yes	18	38.3	29	61.7	0.45	0.23-0.87	0.017^a^
No	85	58.2	61	41.8			
**Megacolon**							
Yes	9	64.3	5	35.7	1.63	0.53-5.05	0.395^a^
No	94	52.5	85	47.5			
**Dyslipidemia**							
Yes	15	75.0	5	25.0	2.90	1.01-8.32	0.041^a^
No	88	50.9	85	49.1			
**Diabetes mellitus**							
Yes	6	54.5	5	45.5	1.05	0.31-3.57	0.936^a^
No	97	53.3	85	46.7			

SD: standard deviation; Group A: patients with heart failure secondary to Chagas disease; Group B: patients with Chagas disease free of systolic dysfunction; bpm: beats per minute; OR: odds ratio;

a chi-square test;

b unpaired t-test;

c Mann Whitney test

All patients with HF were receiving drug treatment and 73.2% were smokers, which was statistically different from group B (p = 0.004). With respect to the comorbidities associated with HF, there was a predominance of dyslipidemia (75%). Mean heart rate was higher in HF patients (p = 0.030) as compared with group B.

Megaesophagus was prevalent in group B only (61.7%), with significant difference between the groups (p = 0.017). The time elapsed since the diagnosis of Chagas disease was also statistically different between the groups (p = 0.001).

### Genetic profile of the study population

In order to determine the prevalence of ACE polymorphism genotype between groups A and B, we analyzed the frequency of the DD, ID and II genotypes (Table 2). There was no statistically significant difference in the observed-to-expected genotype frequencies between the groups (0.692).

**Table 2:** I/D polymorphism in groups A and B

Genotype	Group A		Group B		p-value
	N	%	N	%	
DD	17	50.0	17	50.0	0.692**^a^**
ID	59	56.2	46	43.8	
II	27	50.0	27	50.0	

DD: deletion/deletion; ID: insertion/deletion; II-insertion/insertion; group A: patients with heart failure secondary to Chagas disese; Group B: patients with Chagas disease without systolic dysfunction.

a chi-square test;

Mean values of echocardiographic variables and genotypes were not statistically different between the groups. ID genotype carriers had greater mean LVDD as compared with other genotype carriers.

With respect to repeated measures of categorical data, there was no significant difference in functional class or I/D genotype (p = 0.472) between the groups. There were only four patients in functional class IV; functional class II was present in 86 patients, 52.3% of them belonged to ID genotype.

Megaesophagus was present in group B, with no difference in the number of patients with and without megaesophagus. Dyslipidemia was associated with a 5-time increased risk for HF patients. Genotypes DD, ID and II were not considered as a risk factor for HF, since their distribution was not statistically different between the groups.

## Discussion

There are many conflicting results in the literature on what polymorphisms are involved in the susceptibility to the development and worsening of HF. In the present study, the role of ACE gene polymorphism (I/D) in patients with chagasic heart disease and in Chagas disease patients free of systolic dysfunction. In this population, ACE polymorphism was not associated with sociodemographic and clinical characteristics.

Male gender was predominant (63%) in our sample, similar to data reported in the literature.^9,10^ The incidence of HF increases with age, and is more frequent among men.^11^ The epidemic increase in HF among the older population has been associated with improved survival.^12^

There was no statistically significant difference in the genotype distribution between men and women in group A, which is in accordance with the study by Zhang et al.^13^

Current literature suggests an association of allele D with predisposition to HF,^14-16^ which is in disagreement with our findings. HF patients had lower blood pressure than patients with Chagas disease without systolic dysfunction (p = 0.000), which is in agreement with the study by Yang et al.^17^ who investigated ACE I/D genotype in a Chinese population. There were no significant differences in the frequency of alleles or genotypes between the groups in both sexes. HF patients with low blood pressure are at higher risk of death, despite adequate drug therapy.^18^

An independent association has been reported between DD genotype and worse echocardiographic outcomes, and between ID genotype and echocardiographic profile (increased left ventricular ejection fraction and decreased left ventricular diameters).^19^ These findings are in disagreement with ours, as we did not find an association between D/I genotypes and echocardiographic findings.

In our study, although we investigated a population with different characteristics, no interaction between I/D and HF was found. This is in accordance with a previous study^20^ including 241 patients in Saudi Arabia, in which ACE gene polymorphism was not associated with congenital heart disease.

HF is a common clinical condition with high morbidity and mortality rates. It affects 1.5-2.0% of the general population, and its prevalence increases with age, affecting approximately 10% of individuals aged over 65 years.^21^ These data corroborate our findings, which showed that patients with HF were significantly older than patients with Chagas disease without systolic dysfunction.

In addition, Yang et al.^22^ compared the distribution of I/D genotypes in 701 individuals of both sexes. No difference was found in the frequencies of genotypes and alleles in male and female between individuals aged over 90 years and a control group aged less than 60 years.

In the analysis of I/DI genotypes and LVSD, we did not find any relationship between these parameters. This is in disagreement with a national study^23^ reporting increased LVSD in DD genotype patients, which was associated with increased mortality and morbidity in HF patients of different etiologies.

There was a possible interaction between ACE polymorphisms in chronic HF progression.^24^ Allele D was associated with HF progression and higher mortality rate as compared with allele I.^24,25^ These data are in contrast to our results, in which I/D genotypes were not associated with HF severity.

In our study, ACE polymorphism was not associated with the severity or progression of HF secondary to Chagas disease. This is in agreement with previous studies^26,27^ in which ACE polymorphism was not associated with HF development or progression of Chagas cardiomyopathy.

Distribution of I/D genotypes was not different between groups A and B in our analysis. Individual genetic differences may lead to different risk profiles and small sample sizes, particularly in studies of association, with inadequate power to detect genetic contributions, which may explain the disagreement between studies.

DNA analysis tests may provide the identification of one or more genetic variants associated with increased risk for HF, and thereby contribute to preventive measures including changes in lifestyle and therapies that take into account the genetic profile.

Based on the potential use of the genetic marker in the clinical practice and the inconclusive results regarding the role of ACE polymorphism as a risk factor for the development of HF secondary to Chagas disease, this genetic marker was shown not to be useful in the clinical practice. The lack of association between I/D genotypes may indicate that ACE polymorphism does not act in the pathogenesis of ventricular dysfunction caused by Chagas disease.

## Conclusion

There was no difference in the frequencies of I/D genotypes in patients with HF secondary to Chagas disease as compared with Chagas disease patients free of systolic dysfunction. No relationship was found between ACE polymorphism and clinical outcome measures.

### Study limitations

The number of patients included in the present study may be considered small as compared with the estimated number of patients with Chagas disease in our country. Socioeconomic factors may interact with genetic factors and affect HF outcomes. Our findings were obtained from public health patients, which may limit the extrapolation of the results to other populations.

Further large, prospective studies involving larger samples sizes are needed to determine which variables may be related to HF secondary to Chagas disease.
